# Effects of specific allergen immunotherapy on biological markers and clinical parameters in asthmatic children: a controlled-real life study

**DOI:** 10.1186/s12948-017-0064-5

**Published:** 2017-04-03

**Authors:** I. Djuric-Filipovic, Marco Caminati, D. Filipovic, C. Salvottini, Z. Zivkovic

**Affiliations:** 1 Faculty of Medical Science, University of Kragujevac, Svetozara Markovica 64, 34000 Kragujevac, Serbia; 2 Allergy Unit and Asthma Center, Verona University and General Hospital, Piazzale Stefani 1, 37126 Verona, Italy; 3 Institution for Emergency Medical Care, Bulevar Franša Depera 5, 11000 Belgrade, Serbia; 4 Department of Internal Medicine and Therapeutics, University of Pavia, Strada Nuova 65, Pavia, Italy; 5 Children’s Hospital for Lung Diseases and Tuberculosis, Medical Center “Dr. Dragiša Mišović”, Belgrade, Pilota Mihajla Tepica 1, 11000 Belgrade, Serbia; 6 Faculty of Pharmacy, University Business Academy in Novi Sad, Trg Mladenca 5, 2100, Novi Sad, Serbia

**Keywords:** Allergen specific immunotherapy, Asthma, Childhood, exhaled NO

## Abstract

**Background:**

Allergen-specific immunotherapy (AIT) is the only treatment able to change the natural course of allergic diseases. We aimed at investigating the clinical efficacy of SLITOR (Serbian registered vaccine for sublingual allergen specific immunotherapy).

**Methods:**

7–18 years old children with allergic asthma and rhinitis were enrolled and addressed to the active (AIT plus pharmacological treatment) or control (standard pharmacological treatment only) group. Clinical and medications scores, lung function and exhaled FeNO were measured at baseline and at every follow-up.

**Results:**

There was a significant improvement in both nasal and asthma symptom scores as well as in medication score in SLIT group. SLIT showed an important influence on lung function and airway inflammation.

**Conclusions:**

Our data showed that SLITOR was effective not only in terms of patient reported outcomes but an improvement of pulmonary function and decrease of lower airway inflammation were also observed.

## Background

Asthma is a chronic disease of the airways characterized by inflammation and bronchial remodeling. With a global prevalence of 9.4% in 6–7 years old patients and 12.6% in 13–14 years old patients asthma is one of the most common chronic diseases in childhood age [1, 2]. The growing medical and social burden of asthma is often described as the ‘allergy epidemic’ [3]. Allergen-specific immunotherapy (AIT) holds a great promise in the management of allergic conditions, as it is the only treatment able to change the natural course of respiratory allergic diseases [4]. The disease modifying effect assumes a special relevance in the pediatric age, when the plasticity of the immune system is maximal, and the preventive effects can be reasonably expected [5]. During the last three decades sublingual immunotherapy (SLIT) impressively developed, offering patients an excellent safety and acceptance profile, and a similar efficacy profile when compared with subcutaneous immunotherapy (SCIT) [6, 7]. Although the clinical efficacy of SLIT in children with asthma and allergic rhinitis has been proved in many double blind placebo control randomized clinical trials (DBPC-RCT) and meta-analysis, there is a lack of objective measures related to SLIT efficacy, besides patients reported outcomes [8]. Most of the published studies have considered clinical scores as the main efficacy parameter, whilst immunological and inflammatory parameters have been only occasionally investigated [9]. Recent research has been more focused on identifying objective biomarkers. They can be helpful in early detection of subjects at risk of asthma development as well as in asthma management, from the diagnosis to follow-up, and in treatment tailoring [10]. Up to now several immunological changes related to AIT mechanisms of action have been described: allergen specific IgE, allergen-specific blocking IgG4, eosinophil reactivity, FeNO, eosinophil cationic protein (ECP), allergen specific suppressor T cells as well as the deviation of type 2 T helper cells (Th2) response in favor of Th1 response [11, 12]. FeNO measurement is currently the only validated non-invasive method for assessing asthma-related eosinophilic inflammation in clinical practice. Literature data has already shown that treatment with inhaled or oral corticosteroids as well as with biological treatment such as monoclonal humanized anti-IgE antibody is able to decrease the level of FeNO in children with asthma and allergic rhinitis [13].

The aims of our study were:

1. To prove the clinical efficacy of SLITOR (registered vaccine for sublingual allergen specific immunotherapy) produced by the local Serbian Institute for virology, vaccines and serum (Torlak, Belgrade, Serbia) in terms of improvement of clinical symptoms (nasal and bronchial symptoms) and decrease of medication usage.
2. To show the impact of SLIT on the improvement of pulmonary function
3. To investigate the influence of SLIT on eosinophil airway inflammation—measured with the concentration of exhaled NO (FeNO)

## Methods

Our study was a real life controlled observational study. The study was conducted in the Children’s Hospital for Lung Diseases and Tuberculosis, Medical Centre “Dr Dragiša Mišović”, Belgrade, Serbia. The protocol was approved by the Ethical Committee of the hospital. Informed consent was obtained from all parents or caregivers of the participants. The active group was addressed to SLIT plus standard pharmacotherapy, whereas the control group undertook standard pharmacological treatment only.

Patients were considered eligible for SLIT according to the following factors: diagnosis of allergic rhinitis, diagnosis of asthma under control with standard pharmacological treatment (without acute exacerbation in the last 6 months, without systemic corticosteroids in the last 6 months, without hospitalisation due to acute asthma attacks in the last 6 months, FEV1 ≥80%), positive skin prick tests with inhaled allergens, positive in vitro tests (CAP-RAST immunoassay, minimum IgE class III), age range between 7 and 18 years old. Hypersensitivity to any of the vaccines components, presence or suspect of malignancies, autoimmune systemic diseases as well as immunodeficiency were considered exclusion criteria.

Skin prick tests (SPT) were performed according to published guidelines with a standard battery of glycerinated extracts (Institute of Virology, Vaccines and Sera TORLAK, Belgrade, Serbia). The following allergens were tested: house dust, dust mite (*Dermatophagoides* spp.), cockroach, mold, animal dander, pollens (tree, grass and weed). Histamine and saline were used as positive and negative controls, respectively. A drop of each allergen extract was placed on the volar surface of the forearm and was penetrated with a separate lancet. After 15 min, the wheal reaction was measured as the mean of the longest diameter and the diameter perpendicular to it. Reactions (mean wheal diameter ≥3 mm) were considered positive [17]. Serum specific IgE to allergens extract were assayed with an automated immuno-fluorimetric method (ImmunoCAP 100; Phadia, Upsalla, Sweden). The results were expressed as CAP scores from class 0–6, according to the manufacture’s instruction, (≥class 3 was accepted as relevant).

**Fig. 1:**
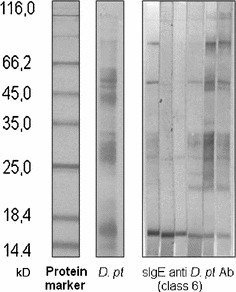
Pre-treatment SDS-PAGE analysis of *Dermatophagoides pteronyssinus* extract and IgE-binding profiles class 6 from six sIgE positive patients from SLIT group. *SDS*-*PAGE* sodium dodecyl sulfate–polyacrylamide gel electrophoresis, *D. pt Dermatophagoides pteronyssinus*, *Ab* antibody, *sIgE* specific immunoglobulin E, *SLIT* sublingual immunotherapy

SLITOR (registered vaccine for sublingual allergen specific immunotherapy) produced by the local Serbian Institute for virology, vaccines and serum Torlak, Belgrade, Serbia was used in the study. The allergen extracts were used for the preparation of sublingual-swallow “vaccines” in phosphate-buffered saline with 50% glycerol. Quality of allergen extract was tested with sodium dodecyl sulphate polyacrylamide gel electrophoresis (SDS-PAGE) and western blot technique (Fig. 1). The potency of the solution was expressed as protein nitrogen unit (PNU)/ml and prepared in three strengths: 16, 125 and 1000 PNU/ml.

According to manufacturer’s recommendations, in the build-up phase (45 days), patients received increasing doses of the extract, starting with one drop of 16 PNU/ml and increasing to 15 drops in 15 days. Daily dose was taken sublingually, applied on a sugar cube in the morning, half an hour before breakfast. This process was repeated also for the 125 and 1000 PNU/ml. Finally, patient was switched to maintenance phase regimen, using 15 drops of the 1000 PNU/ml twice a week for the following 24 months. Allergen proteins concentration in maintenance therapy was equivalent to 19.9 μg/ml i.e. 0.995 μg of allergen proteins in one drop of extract. Calculated mean cumulative monthly dose of allergen proteins was 119.4 μg, while the mean cumulative dose per year was about 1.4 mg.

Patients from both groups (irrespective to SLIT) received an appropriate pharmacological treatment according to ARIA and GINA guidelines depending on symptoms: oral antihistamines, intranasal corticosteroid, inhaled corticosteroid and inhaled bronchodilator.

## Clinical evaluation

All patients were followed up during the 2 years from the beginning of the protocol. Patients were asked to fill in the symptom and medication score diary on a daily base twice a day (in the morning and in the evening) during 1-month period or during the pollen season for patients who were sensitized to seasonal allergens. Older children were also asked to calculate the mean values, usually with parents help.

The following symptoms of AR were scored: rhinorrhea, sneezing, nasal itching and blocked nose. In addition, for the AR with AA patients, next symptoms were scored: chest tightness, shortness of breath, cough and wheezing. Each symptom was scored as 0 (absent), 1 (mild), 2 (moderate), 3 (severe) and the mean monthly symptom score (SS) was calculated. The use of symptomatic medications was also recorded daily, during the same period. Anti-allergic medication requirement was evaluated as the monthly mean medication score (MS). Airways eosinophilic inflammation measurement was performed with NIOX MINO (Aerocrine, Solna, Sweden). The data were interpreted according to the recommendations of American Thoracic Society (ATS) [14–17] (Table 1). Conditions potentially influencing FeNO values (anxiety, cardiac disease, chronic obtrusive disease, GERB, non eosionophilic asthma, rhinosinusitis, voice cord dysfunction, cyctic fibrosis, primary ciliary dyskinesia infection and asthma exacerbation) were excluded.

**Table 1:** FeNO interpretation

Asthma	FeNO (>12 years) (ppb)	FeNO(<12 years) (ppb)
Control asthma	<25	<20
Intermediate	25–30	25–30
Non-control asthma	>50	>35

Lung function test was performed at each visit using Jaeger, Pneumo Screen spirometry. Subjects were advised to avoid the use of the short-acting bronchodilator at least 12 h before the test. FEV1 values were expressed as a percentage of predicted values.

The patients receiving SLIT were required to record and give their report on a specific diary card, in the case of side effects: local (oral itching/burning, swelling, oedema of the uvula or tongue) or systemic adverse reactions (asthma, rhinitis, urticaria, angioedema, generalized itching, gastrointestinal symptoms—abdominal pain, nausea, vomiting, shock).

## Statistical analysis

The sample size was calculated with the software package G power. A sufficient number of observation units for the error level α = 0.05 and power of the study 1−β = 0.8 is 0.72 were considered. Descriptive and analytical statistical methods were used. The following descriptive variables were described: measures of central tendency (mean, median), measure of dispersion (standard deviation, interval of variation). Analytical statistical methods were used to test differences, parametric and nonparametric variables. Student’s t test and analysis of variance of repeated measurements were used. Chi square test, McNemar test, Mann–Whitney test, Wilcoxon test, Friedman test were also included. All data were analyzed in SPSS 15.0 software package. (SPSS Inc., Chicago, Illinois, USA).

## Results

Overall 59 patients (mean age, 13.18 ± 3.433 range 7–20 years; 50.8% boys; 49.2% girls) were included: 34 (20 girls and 14 boys) received SLIT as an add-on to drug therapy and 25 (10 girls and 15 boys) received anti-allergic and asthmatic drug therapy alone. Patients from SLIT and control group were homogenous for all demographic and clinical characteristics.

We found clinical improvement in the SLIT group, demonstrated by statistically significant decrease of all rhinitis symptoms after 2 years of SLIT vs. baseline for both groups (Table 2).

**Table 2:** The distribution of values for a patient rhinitis symptom scores

Nasal congestion	χ^2^ = 37,783; p < 0.001
Nasal pruritus	χ^2^ = 38,346; p < 0.001
Rhinorrhea	χ^2^ = 42,012; p < 0.001
Sneezing	χ^2^ = 44,831; p < 0.001

According to our statistical analysis 75% of patients on SLIT didn’t complain about nasal congestion after 2 years of treatment; 80% of the patients in the same group didn’t have nasal pruritus, whereas SLIT was effective in treating rhinorrhea and sneezing in 75% patients. On the other side standard pharmacotherapy didn’t have such a significant impact on nasal symptoms. We also found a statistically significant inter-group difference for all rhinitis symptom scores, after 1st year with a further improvement of symptoms in the group on SLIT during the 2nd year of follow up period.

A similar clinical improvement SLIT expressed on asthma symptom scores. Our results demonstrated decrease of all asthma symptom scores during the follow up period for all participants with a statistical significant influence of SLIT group on that improvement. The results are showed in Table 3. After 2 years of SLIT treatment more than 80% of the patients didn’t complain about cough, night cough, and chest breathless and wheezing. We also found a statistically significant inter-group difference for all asthma symptom scores, after 1st year with a further improvement of symptoms in the group on SLIT during the 2nd year of follow up period. All data are summarized in Table 4.

**Table 3:** The distribution of values for asthma symptom scores

Cough	χ^2^ = 62,384; p < 0.001
Night cough	χ^2^ = 47,743; p < 0.001
Chest breathless	χ^2^ = 49,622; p < 0.001
Wheezing	χ^2^ = 49,078; p < 0.001

**Table 4:** χ^2^ test symptoms scores during SLIT course

Symptoms	At the beginning χ^2^	After 1 year χ^2^	After 2 year χ^2^
Nasal congestion	10,299	8732	10,835
Nasal pruritus	5601	8877	8737
Rhinorrhea	8119	10,001	12,464
Sneezing	6407	10,605	9821
Cough	8100	5322	16,028
Night cough	9114	5177	12,666
Chest breathless	5656	2154	9680
Wheezing	10,664	12,294	12,362

χ^2^ test showed statistical significant differences for all rhinitis and asthma scores in all of three periods. At the beginning of the follow up period children in SLIT group had more severe symptoms in comparison with children on standard pharmacotherapy. Even after a 1-year follow up we, a significant improvement was registered. Similar results were observed at 2nd year, especially for patients with more severe symptoms.

**Fig. 2:**
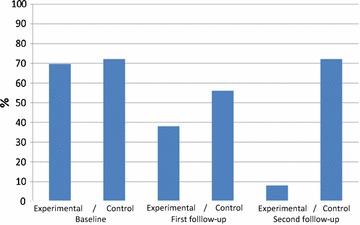
The usage of β2 agonists

The data from our study showed that after 2 years the use of inhaled corticosteroids, intranasal corticosteroids, β2 agonists was significantly reduced in the group of patients on SLIT (Z = − 4311 p < 0.001, χ^2^ = 30,785; p < 0.001, Q = 28,783; p < 0.001 respectively), in comparison with the control group (Fig. 2). The patients in the experimental group also used statistically less antihistamines (χ^2^ = 32,774; p < 0.001) and leukotrienes (*χ^2^ = 30,785; p < 0.001) in comparison with the patients in non-SLIT group, but only after 2 years of AIT.

**Fig. 3:**
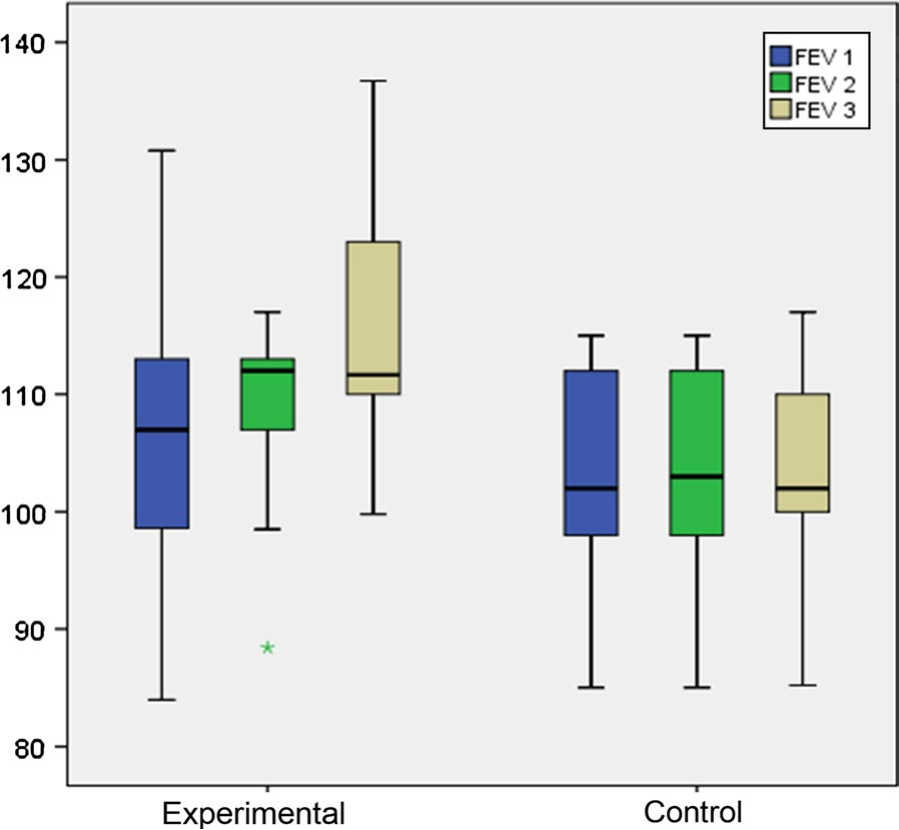
Respiratory function

Although at the beginning of the study all patients had FEV1 ≥80% of predicted value, SLIT showed a significant improvement of FEV1 just after 1 year with a further improvement in the 2nd year of follow up period (F = 3514; p = 0.036), while at the same time FEV1 remain without any improvement in children on standard pharmacotherapy (F = 3199; p = 0.048) (Fig. 3).

**Fig. 4:**
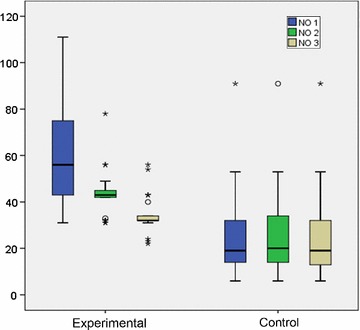
The level of FeNO

The level of FeNO decreased significantly in all the three measurements during SLIT course (χ^2^ = 52,220; p < 0.001). During the follow up period significant differences between the groups in all three measurements were observed. Patients in the experimental group had significantly higher values of FeNO in all measurements. When we compared the values in each group independently, we found only significant reduction in the experimental group. We observed both significant reduction between FeNO1 and FeNO2 (p < 0.001) and between FeNO2 and FeNO3. Throughout the treatment period there was a sustained significant reduction between FeNO2 and FeNO3. Max value for FeNO1 was 111 ppb, while MaxFeNO2 and FeNO3 were 78 and 56 ppb consecutively. Here we showed that there is an influence of SLIT on the FeNO values in children in experimental group, whereas no reduction in FeNO values were registered in the control group (Table 5, Fig. 4).

**Table 5:** The level of FeNO (Legend FeNO I baseline. FeNO II–1st year of follow-up period, FeNO III-2nd year of follow-up period)

	N	Mean value	SD	Median	Minimum	Maximum
No 1	34	60.65	20.467	56.00	31	111
No 2	34	43.18	8.990	43.00	31	78
No 3	34	34.15	6.985	32.00	22	56

### Safety assessment

There were no local side effects that required treatment. Three side effects were reported. All of them involved mild to moderate gastrointestinal complaints (mouth burning or itching and stomachache and nausea) and self- resolve in a few days without any intervention. It is noteworthy that no serious adverse events were reported in the present survey, and the incidence of side-effects appeared to be lower than that reported for injective immunotherapy.

## Discussion

This is one of the first studies evaluating clinical efficacy, pulmonary function, FeNO and safety in children with both allergic rhinitis and asthma undergoing SLIT or drug treatment. In the current study we found: (a) statistically significant improvement in nasal and asthma symptom scores after 1 year of treatment in SLIT group, with further improvement in the 2nd year of follow up (75 and 80% respectively) compared with control group; (b) only three patients reported mild local and systemic adverse reactions; (c) statistically significant improvement of pulmonary function; (d) statistically significant decrease of eosinophilic inflammation of lower airways measured with FeNO. Since SLIT was first introduced for treatment of respiratory allergies in children and later accepted as a viable alternative to SCIT, need for an assessment of its efficacy and safety in respiratory allergy has emerged [18]. Consequently, many randomized double blind placebo controlled and open controlled trials [19–27], as well as a number of systematic reviews and meta-analyses have been carried out to determine the efficacy and safety of SLIT [28–36]. Data from literature suggested overall clinical effectiveness of SLIT in patients with AR and AA, although the conclusions were restricted by heterogeneity of the studies, especially concerning the manufacturer’s variability of allergen content in commercial extracts. In our study the only available SLIT extracts in Serbia was used, and the exact dose in micrograms of major allergen was calculated.

The comparison between immunotherapy and standard pharmacological treatment is still a matter of debate. Clinical effects of SLIT can be appreciated only in the long term period (months), whereas traditional drugs act immediately. Data from literature showed that efficacy of SLIT is dose-dependent and sufficient duration of treatment is essential to elicit the immunologic changes underlying its clinical effectiveness. According to our results, SLIT seems to be a beneficial therapeutic strategy. In our study, after 12 months of treatment, reduction in all clinical scores was observed in the SLIT group (in up to 75% of the patients). Reduction of drug intake indicates that pharmacological treatment does not prime SLIT efficacy. Comparison between groups showed statistically significant reduction of drug scores and symptoms scores in SLIT group.

Exhaled NO has been shown to reflect the levels of airway inflammation in asthmatic patients [37, 38]. In addition, it has also been reported that asthmatic patients show higher levels of NO in peripheral blood and that serum levels can be used as an additional inflammatory marker in asthma [39]. No study has yet investigated the effect of AIT on NO concentration, although AIT with *D. pteronyssinus* and *D. farinae* extracts has been found to reduce exhaled NO in asthmatic children with mite allergy [40]. However the results from the studies are controversial and a clear demonstration of a reduction in exhaled NO in asthmatic patients taking SLIT is lacking [41]. According to our data NO levels decreased after SLIT, possibly reflecting a reduction in systemic allergic inflammation.

Some potential limitations of our study have to be pointed out. Patients were not stratified according to sensitizations, which could have an impact on SLIT effectiveness and results of clinical scores. The small study population sample did not allow a proper subanalysis by sensitization profile. Furthermore the quality of SLIT extract is questionable, but the extract we used is the only available product in Serbian market.

## Conclusions

Our data showed that SLITOR is an effective treatment, decreasing both symptom and medication scores in the active group. These findings suggest that SLIT may have preventive effects, showing in children with intermittent asthma and AR a lower occurrence of persistent airway inflammation. Combining clinical outcomes with respiratory function and FeNO values, we could be able to phenotype the most adequate patients who will benefit from SLIT. When evaluating the effect of AIT, it is appropriate to consider results affecting both the upper and the lower airways, whereas measurement of FeNO is of a great importance.

## Abbreviations

AIT: allergen-specific immunotherapy
DBPC-RCT: double blind placebo control randomized clinical trials
ECP: eosinophil cationic protein
FeNO: exhaled nitric oxide
FEV1: forced expiratory volume in 1 s
GERD: gastro-esophageal reflux disease
GINA: Global initiative for asthma
ICS: inhaled corticosteroids
INCS: intranasal corticosteroids
ITT: intent-to-treat
LABA: long acting beta agonists
RAST: radioimmunoassay allergen specific test
SCIT: subcutaneous immunotherapy
SLIT: sublingual immunotherapy
SPT: skin prick test
